# Pancreatic cancerrelated cachexia: influence on metabolism and correlation to weight loss and pulmonary function

**DOI:** 10.1186/1471-2407-9-255

**Published:** 2009-07-28

**Authors:** Jeannine Bachmann, Knut Ketterer, Christiane Marsch, Kerstin Fechtner, Holger Krakowski-Roosen, Markus W Büchler, Helmut Friess, Marc E Martignoni

**Affiliations:** 1 Department of Surgery, Klinikum rechts der Isar, Technische Universität München, Munich, Germany; 2 Department of General Surgery, University of Heidelberg, Heidelberg, Germany; 3 Department of Radiology, University of Heidelberg, Heidelberg, Germany; 4 German Cancer Research Center, Heidelberg, Germany

## Abstract

**Background:**

Dramatic weight loss is an often underestimated symptom in pancreatic cancer patients. Cachexia- defined as an unintended loss of stable weight exceeding 10% – is present in up to 80% of patients with cancer of the upper gastrointestinal tract, and has a significant influence on survival. The aim of the study was to show the multiple systemic effects of cachexia in pancreatic cancer patients, in terms of resection rate, effects on pulmonary function, amount of fat and muscle tissue, as well as changes in laboratory parameters.

**Methods:**

In patients with pancreatic cancer, clinical appearance was documented, including the amount of weight loss. Laboratory parameters and lung-function tests were evaluated, and the thickness of muscle and fat tissue was measured with computed tomography scans. Statistical analysis, including multivariate analysis, was performed using SPSS software. Survival curves were calculated using Kaplan-Meier analysis and the log-rank test. To test for significant differences between the examined groups we used Student's t-test and the Mann-Whitney U test. Significance was defined as p < 0.05.

**Results:**

Of 198 patients with a ductal adenocarcinoma of the pancreas, 70% were suffering from weight loss when they presented for operation, and in 40% weight loss exceeded 10% of the stable weight. In patients with cachexia, metastases were diagnosed significantly more often (47% vs. 24%, P < 0.001), leading to a significantly reduced resection rate in these patients. Patients with cachexia had significantly reduced fat tissue amounts. Hence, dramatic weight loss in a patient with pancreatic cancer may be a hint of a more progressed or more aggressive tumour.

**Conclusion:**

Pancreatic cancer patients with cachexia had a higher rate of more progressed tumour stages and a worse nutritional status. Furthermore, patients with cachexia had an impaired lung function and a reduction in fat tissue. Patients with pancreatic cancer and cachexia had significantly reduced survival. If weight loss exceeded 5% there was a significantly reduced resection rate to detect, but the changes were significantly more substantial if weight loss was 10% or more. We propose that a weight loss of 10% be defined as significant in pancreatic cancer.

## Background

Pancreatic cancer is the fourth leading cause of cancer-related deaths in Western countries [1,2]. In 2006, an estimated 32,000 deaths were related to pancreatic cancer in the United States (U.S.) [3]. More than 90% of pancreatic cancers are ductal adenocarcinomas, which often present with perineural and retroperitoneal infiltration. An early diagnosis is necessary, and after the exclusion of metastatic disease, surgical resection of the tumour is the patient's only hope for cure [1,4-10]. Resection offers a significantly improved prognosis, with a median survival of 14–20 months and up to 25% 5-year survival rates. However, most patients have advanced disease at the time of presentation, with various signs and symptoms, such as bile duct obstruction, abdominal pain, loss of appetite, or vomiting [11].

A dramatic weight loss, with subsequent complications, is an often-underestimated symptom, present in two-thirds of the patients with malignant tumors [11-14]. Cancer patients who have lost weight have a poorer prognosis than patients with stable weight [14,15]. A recent study with patients suffering from pancreatic cancer demonstrated, that weight stabilisation was associated with longer survival [16].

The loss of muscle and fat- tissue is mediated by different alterations in the organism, not all of which are known until now [17]. In recent years there has been a major effort to identify the mechanisms underlying muscle and fat degradation in cancer cachexia. One important mechanism is the activation of the acute phase response cascade, resulting in loss of fat and muscle tissue. In patients with pancreatic cancer and an activated acute phase response cascade, weight loss is accelerated, leading to significantly shorter survival [18,19]. Both, increased muscle-protein degradation and reduced muscle-synthesis result in a net loss of muscle tissue [17,20]. Subsequently, pancreatic cancer patients often have an elevated resting energy expenditure (REE) [21]. Additionally, the oral food intake in these patients is lower. This results in a lean body mass depletion. For muscle degradation, the ubiquitin-proteasome pathway is the most important mechanism [22]. Agustsson and coworkers found that in patients with cancer cachexia, adipocyte lipolysis is activated by a hormone-sensitive lipase [23].

Cachexia, defined as an unintended weight loss of 10% or more of the stable weight over a period of 6 months, is present in up to 80% of patients with cancer of the upper gastrointestinal tract [24-26]. A cachectic status has an influence on survival, 30- day- mortality and morbidity [12].

Up to 80% of patients with cancer of the gastrointestinal tract suffer from dyspnea [27,28]. Frequency and severity of dyspnea increase with the progression of the disease [27]. In patients with impaired lung function preoperatively (vital capacity less than 50% of the normal level or less than 2 litres), about every fifth patient can be expected to have pulmonary insufficiency postoperatively [29]. Another important parameter of the pulmonary function is the forced expiratory volume in one second; healthy individuals have values over 70% of the vital capacity measured, compared to patients with cancer of the gastrointestinal tract [29].

The aim of this study was to investigate the influence of cachexia on early changes in patients with pancreatic cancer, with a special interest in the systemic effects of cachexia on fat and muscle tissue and on lung function.

## Methods

### Patients

In 198 patients with histologically confirmed adenocarcinoma of the pancreas, who underwent operation in the Department of Surgery, University of Heidelberg, between June 2004 and February 2006, a careful history was taken and lung function tests were performed. Table 1 shows the patients' characteristics in detail. The study was performed according to the guidelines of the Declaration of Helsinki, and each patient was asked to give written informed consent for data collection as well as for publication of data in an anonymous manner. All data and examinations in this study were acquired in clinical routine, therefore an ethical approval was not needed. All data were collected in a prospectively designed database.

**Table 1:** Patient characteristics and results of labwork in patients with and without cachexia

all patients N = 198		without cachexia N = 119 [60.1%]	with cachexia N = 79 [39.9%]	p value
gender	male	67 [56.3]	49 [62]	P = 0.425
	female	52 [43.7]	30 [38]	

**ASA classification**	I	2 [1.7]	1 [1.3]	**P = 0.023**
	II	57 [47.9]	26 [32.9]	
	III	60 [50.4]	50 [63.3]	
	IV	0 [0]	2 [2.5]	

**resection rate **		95 [79.8]	38 [48.1]	**P < 0.001**
**metastases**		29 [24.4]	37 [46.8]	**P < 0.001**

**tumour stage**	UICC II	83 [69.7]	37 [46.8]	**P < 0.001**
	UICC III	7 [5.9]	5 [6.3]	
	UICC IV	29 [24.4]	37 [46.8]	

30-day mortality		4 [3.4]	4 [5.1]	P = 0.561
morbidity		50 [42.0]	36 [46.2]	P = 0.568

**albumin**	[g/l]	44.2 [41.1/46.1]	41.45 [39.5/43.8]	**P < 0.001**
CA19-9	[U/l]	165.0 [41.3/579.0]	308.0 [43.7/1454.0]	P = 0.163
**CrP**	[mg/l]	5.0 [1.0/11.3]	9.4 [3.0/32.8]	**P = 0.002**
**haemoglobin**	[g/dl]	13.3 [12.15/14.35]	12.8 [11.8/13.7]	**P = 0.023**
**protein**	[g/l]	73.9 [71.1/77.4]	72.2 [68.55/75.75]	**P = 0.037**


**resected patients** N = 133		without cachexia N = 95 [71.4%]	with cachexia N = 38 [28.6%]	**P value**

**ASA classification**	I	2 [2.1]	0 [0]	**P = 0.363**
	II	48 [50.5]	17 [44.7]	
	III	2 [47.4]	21 [55.3]	

30-day mortality		2 [2.1]	1 [2.6]	P = 0.854
morbidity		43 [45.3]	36 [46.2]	P = 0.144

**albumin**	[g/l]	44.1 [40.9/45.9]	41.6 [39.5/43.9]	**P = 0.010**
CA19-9	[U/l]	151.0 [34.0/416.0]	203.6 [25.25/721.0]	P = 0.657
CrP	[mg/l]	5.1 [1.0/11.8]	6.6 [2.2/16.2]	P = 0.282
haemoglobin	[g/dl]	13.1 [12.05/14.25]	13.1 [12/13.7]	P = 0.349
protein	[g/l]	73.8 [71.1/77.2]	70.75 [68.6/75.25]	P = 0.053

Tumor size	T3	92 [96.8]	38 [100]	P = 0.270
	T4	3 [3.2]	0 [0]	
grade	G1	4 [4.3]	4 [11.1]	P = 0.166
	G2	57 [61.3]	23 [63.9]	
	G3	32 [34.4]	9 [25.0]	

### Weight and body composition

For every patient the current height and weight were registered; when a patient was suffering from weight loss, the time period was also documented. A patient was classified as cachectic, when he had lost 10% or more of his stable pre-illness weight.

### Histological diagnosis

For each patient two independent pathologists form the Department of Pathology, University of Heidelberg, confirmed the diagnosis. Resected tumours were classified histopathologically according to the TNM classification, version 2005, including examination of the resection margin and grading. Tumour staging was determined according to the UICC classification 2006 [30,31].

### Morbidity

Every complication which prolonged the hospital stay and/or had to be treated (by interventional placing of a drain, reoperation, or non-invasive therapy) was noted. The prevalence of wound infection, postoperative bleeding, and cholangitis, pancreatic fistula, intraabdominal abscesses, delayed gastric emptying, pneumonia, urinary tract infection, myocardial infarction, and pulmonary embolism were evaluated [32,33].

### Pulmonary function tests

Lung function was measured using a spirometer (OPTI PLEX 466le). A standardised protocol was used, and before the test began the procedure was demonstrated to the patient by an independent examiner, who did not know whether the patient had cachexia. In every patient the vital capacity (VC) in absolute and relative values of the expected values as well as forced expiratory volume in one second (FEV1) was documented.

### Measurement of fat and muscle tissue on computed tomography scans

Every patient received a contrast-enhanced CT scan. A venous phase of a computed tomography scan of the abdomen was chosen.

**Figure 1:**
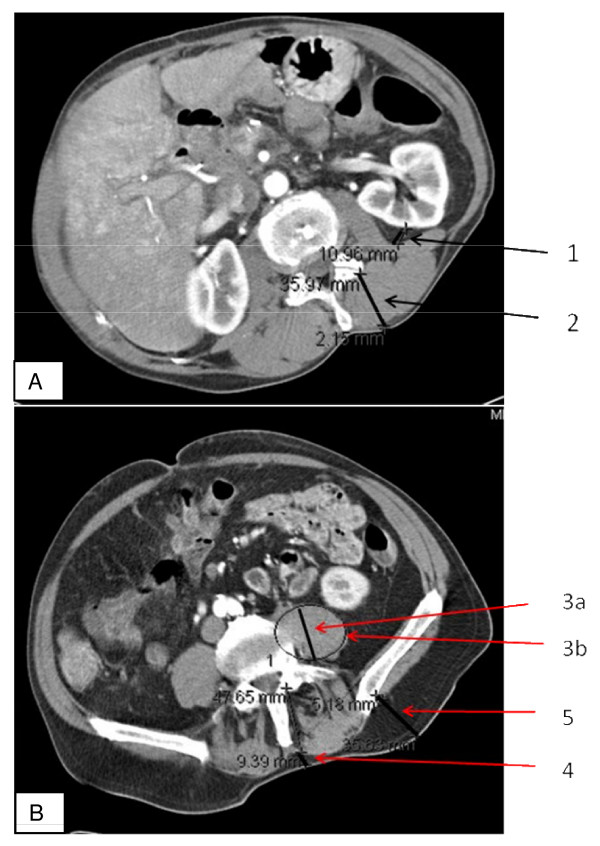
**A: CT slide of renal pelvis**. [1] **perirenal fat**: orthographic to renal capsule and to line 2. [2] **musculus erector spinae**: thickness of the muscle in a parallel line to the dorsal process Figure 1B: CT slide of iliac crest. [3a] **thickness of the musculus psoas **in a parallel line to the dorsal process. [3b] **area of the psoas muscle**. [4] **subcutaneous fat medial**: parallel to the dorsal process. [5] **subcutaneous fat lateral**: parallel to the dorsal process starting at iliac spine posterior superior area of the psoas muscle.

Six different values were taken: (figure 1 shows an example of the measured lengths) the thickness of the perirenal fat [1] and of thesubcutaneous fat [4] and [5]; Thickness of muscle was measured in two different locations–the musculus erector spinae [2] and the musculus psoas [3a]–and area was measured as well [3a].

### Statistical analysis

Statistical analysis, including multivariate analysis, was performed using SPSS software, version 16 (SPSS Inc., Chicago, IL, U.S.). Survival curves were calculated using Kaplan-Meier analysis and the log-rank test. To test significant differences between the examined groups, we used Student's t-test and the Mann-Whitney U test. Significance was defined as p < 0.05. Results are reported as median and lower (lq) and upper (uq) quartiles.

## Results

Of 198 patients (116 men and 82 women; median age 64 [57/79] years) with a histologically proven adenocarcinoma of the pancreas, 69.7% (138) patients presented with weight loss; in 39.9% (79) of the patients, weight loss exceeded 10% of the pre-illness stable weight (table 1), and these patients were therefore categorized as cachectic. In patients without cachexia, the median time period, in which weight loss occurred was 2 [1/4] [lower quartile/upper quartile] months; in patients with cachexia it was 4 [2/6] months (P < 0.001).

In 67.2% (133) of patients a tumour resection could be performed; in the other 32.8% (65) a palliative operation (either an explorative laparotomy or a bypass operation) was performed due to locally advanced tumour growth or distant metastases to the peritoneal cavity or the liver. Table 1 shows the patient characteristics in the patients scheduled for tumour resection; they were divided into a group with and a group without cachexia (table 1).

The resection rate was significantly lower in patients with cachexia (P < 0.001). In 79.8% of patients without cachexia a tumour resection was performed, whereas a resection was only possible in 48.1% of patients with cachexia. Patients with cachexia had significantly progressed tumour stages (P < 0.001, table 1): 24.4% of patients without cachexia had occult distant metastases diagnosed on explorative laparotomy; in contrast, metastases to the peritoneal cavity or the liver were present in 46.8% of patients with cachexia (P < 0.001). There were no significant differences between the two examined groups in 30-day mortality (P = 0.561) and perioperative morbidity (P = 0.568), even when corrected for tumour resection and palliative operation. In this series perioperative course was no worse in patients with cachexia than in those without cachexia.

Blood tests showed significant differences in protein levels (P = 0.037) and albumin levels (P < 0.001), with significantly lower levels in patients with cachexia compared to patients without cachexia, demonstrating a worse nutritional status in cachectic patients. Haemoglobin values were also significantly reduced in patients with cachexia (P = 0.023), without any signs or symptoms of occult bleeding. CA19-9 values, as markers for tumour load, were not significantly different between patients with and without cachexia (P = 0.163), demonstrating that it is not tumour load that is essential but the aggressiveness of tumour biology, represented by the systemic alterations in patients with cachexia. The values for C-reactive protein were significantly elevated in patients with cachexia (P = 0.002). In patients in whom a tumour resection could be performed, there was no significant difference in tumour size (P = 0.270, table 1) or grade (P = 0.166) between patients with and without cachexia.

To illustrate the impact of cachexia on pulmonary function, lung function tests were performed in patients with and without cachexia. In the 150 examined patients there was a significantly worse vital capacity (vc%) in patients with cachexia (P = 0.032); in absolute values there was no significant difference (P = 0.142, table 2). The forced end expiratory volume was not significantly different between the two groups (P = 0.514) as well as in relative values (P = 0.137).

**Table 2:** lung function tests (N = 150) and amount of perirenal fat (N = 175), muscle (N = 123) and subcutaneous fat (N = 123) Median values [lower/upper quartile], p value.

parameter	without cachexia median [lq/uq]	with cachexia median [lq/uq]	p value
VC [l]	3.3 [2.7/3.9]	3.1 [2.4/3.9]	P = 0.142
**VC [%]**	93.0 [80.0/102.0]	84.5 [75.0/97.5]	**P = 0.032**
FEV 1 [l]	2.8 [2.5/3.5]	2.7 [2.3/3.4]	P = 0.514
FEV 1 [%]	100 [88.0/115.0]	98.0 [82.0/108.0]	P = 0.137

perirenal fat [1]	9.0 [5.1/13.5]	8.3 [5.1/11.2]	P = 0.336
M. erector spinae [2]	33.0 [29.2/36.5]	31.5 [26.9/36.3]	P = 0.343
M. psoas [3a]	45.1 [38.1/113.0]	49.3 [40.2/130.1]	P = 0.126
M. psoas area [3b]	1158.3 [854.5/1487.3]	1228.9 [952.7/1484.8]	P = 0.713
**subcut. fat medial ** [4]	14.4 [7.5/22.8]	7.9 [5.5/16.9]	**P = 0.016**
**subcut. fat lateral ** [5]	44.5 [35.1/56.3]	38.0 [27.0/45.9]	**P = 0.007**

In 175 of the patients the thickness of perirenal fat could be assessed with a CT scan. We found no significant difference in perirenal fat thickness between patients with cachexia and without cachexia (table 4). In the lower part of the abdomen, on the iliac crest slides, there was no significant difference between the two groups in any of the examined muscle tissue thicknesses. In contrast, fat tissue thickness levels were significantly reduced in patients with cachexia: subcutaneous fat medial (P = 0.016) and lateral (P = 0.007) (Figure 1, table 2).

**Figure 2:**
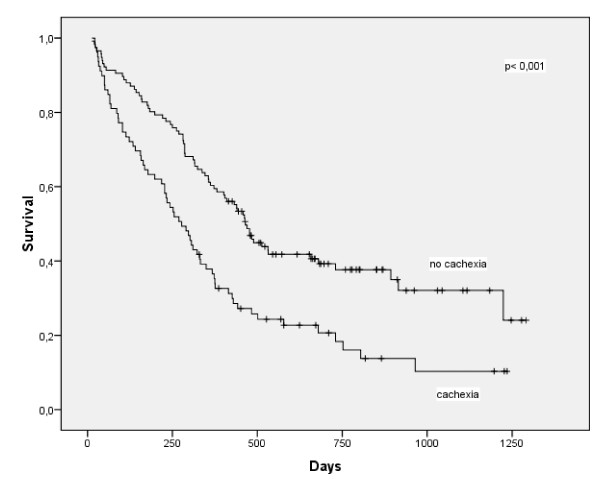
**Kaplan-Meier survival curve in 198 patients with pancreatic cancer with and without cachexia (p < 0.001) showing a significant longer survival in patients without cachexia**.

Patients with cachexia had significantly worse survival than patients without cachexia (figure 2, P = 0.042). When all patients with non-metastasised tumours were compared, patients with cachexia again had shorter survival (P = 0.067), but in UICC stage IV this difference became significant (P = 0.008). In multivariate analysis, tumour stage (P < 0.001) and weight loss (P = 0.003) significantly influenced survival.

To demonstrate that a weight loss of 10% has a significant influence in patients with pancreatic cancer, the evaluation was also performed using a weight loss of 5% as a division parameter. With this separation there was also a significantly worse resection rate in patients suffering from weight loss of 5% or more (P < 0.001), with metastases detected significantly more often on explorative laparotomy in patients with weight loss exceeding 5% (P = 0.006) than in patients with weight loss of less than 5%. Accordingly, in patients with weight loss of 5% or more, the tumour stages were significantly worse (P = 0.011).

In the examined lab work no significant difference could be detected between patients with weight loss of less or more than 5% (table 5). Furthermore, with this separation there was no significant difference in lung function tests, amount of perirenal fat, or muscle thickness (table 3).

**Table 3:** P values for differences in the parameters weight loss </> 5% and, </> 10% respectively

parameter	Weight loss </> 5%		Weight loss </> 10%	
ASA classification	**P = 0.017**	**▼**	**P = 0.023**	▼
resection rate	**P < 0.001**	**▼**	**P < 0.001**	▼
metastases	**P = 0.006**	**▼**	**P < 0.001**	▼
tumour stage	**P = 0.011**	**▼**	**P < 0.001**	▼
30-day mortality	P = 0.925	▬	P = 0.561	▬
morbidity	P = 0.988	▬	P = 0.568	▬

albumin	P = 0.054	▬	**P < 0.001**	▼
CA19-9	P = 0.383	▬	P = 0.163	▬
CrP	P = 0.116	▬	**P = 0.002**	▼
haemoglobin	P = 0.595	▬	**P = 0.023**	▼
protein	P = 0.089	▬	**P = 0.037**	▼

VC [l]	P = 0.283	▬	P = 0.142	▬
VC [%]	P = 0.161	▬	**P = 0.032**	▼
FEV 1 [l]	P = 0.118	▬	P = 0.514	▬
FEV 1 [%]	P = 0.731	▬	P = 0.137	▬

perirenal fat [1]	P = 0.955	▬	P = 0.336	▬
M. erector spinae [2]	P = 0.790	▬	P = 0.343	▬
M. psoas [3a]	P = 0.449	▬	P = 0.126	▬
M. psoas area [3b]	P = 0.243	▬	P = 0.713	▬
subcut. fat medial [4]	**P = 0.004**	▼	**P = 0.016**	▼
subcut. fat lateral [5]	P = 0.063	▬	**P = 0.007**	▼

Patients with weight loss of less than 5% had significantly better survival when a curative resection could be performed (UICC II, P < 0.001). When metastases were detected on explorative laparotomy there was no significant difference in survival between patients with and without cachexia. In patients with cachexia survival was reduced even after curative resection (P = 0.067); survival was dramatically reduced in cachectic patients who had undergone palliative operations (P = 0.008).

Even after curative resection, survival was significantly reduced in patients suffering from weight loss of 5% or more (P < 0.001) in comparison to patients with less than 5% weight loss. In contrast, when metastases to the liver or peritoneal cavity were detected, the division parameter of 5% did not show any significant difference. However, when weight loss exceeded 10% in patients with metastases, survival was significantly reduced.

## Discussion

In this study of 198 patients with a ductal adenocarcinoma of the pancreas we assessed the prevalence of cachexia and its systemic effects. To show an influence of cachectic status on lung function tests and muscle and fat tissue in patients before pancreatic surgery, corresponding data were acquired in selected areas using CT scans. Furthermore, we studied the influence of a dramatic weight loss on postoperative survival.

This study found that 70% of the patients who presented for pancreatic cancer resection were suffering from weight loss, and that in 40% of these patients this loss of weight exceeded 10% of their stable pre-illness weight. These findings demonstrate that even in selected patients who are scheduled for pancreatic cancer resection, nearly 40% present with a significant preoperative weight loss and only 30% do not suffer from weight loss at the time of tumour resection. In 67% of all patients, a tumour resection could be performed: the resection rate in patients with cachexia was significantly worse, due to more frequent diagnosis of metastases to the liver and/or peritoneal cavity. A dramatic weight loss in a patient with pancreatic cancer can be a hint of a more progressed and aggressive tumour.

CA 19-9 is used as a tumour marker in pancreatic cancer. In a study of 176 patients resected for pancreatic cancer, the level of CA19-9 correlated with tumour size[34,35] In our patients there was no difference in preoperative CA 19-9 levels between patients with and without cachexia, which indicates similar tumour sizes in these patients; the more often progressed tumour stage in patients with cachexia thus suggests a more aggressive tumour rather than a larger tumour of the pancreas. These findings are underlined by the fact that no significant differences in tumour size could be seen in patients with and without cachexia after tumour resection. Also with regard to tumour grade, no significant difference between patients with and without cachexia could be detected. This shows that cachexia is not correlated with tumour mass or grade in resectable pancreatic cancer.

Albumin, protein and haemoglobin are serum markers which show the nutritional status in patients with cancer [36]. All three of these markers were significantly reduced in pancreatic cancer patients with cachexia, indicating a worse nutritional status. Despite this worse nutritional status, no significant influence on perioperative morbidity and 30-day mortality was detected in this series. The morbidity and 30-day mortality rates in our patients are consistent with rates in other publications [37].

The cachectic syndrome had a significant impact on the lung function tests in pancreatic cancer patients with cachexia; the relative vital capacity was significantly worse in these patients; despite this finding, the impaired lung function test results had no negative influence on perioperative mortality and morbidity. There was no significant difference between the two groups in the examined muscle thicknesses; despite this finding, the lung function in pancreatic cancer patients with cachexia was significantly reduced, showing that in the early stages of the cachectic syndrome the muscle is preserved, but the function of the muscle is impaired. We could demonstrate a significant influence of cachexia on fat metabolism, leading to significantly reduced fat tissue amounts in pancreatic cancer patients with cachexia; this result underlines the systemic effects of cachexia in patients with pancreatic cancer.

In patients with cachexia the survival was shortened even after curative resection, but survival was dramatically reduced in patients with cachexia undergoing palliative operations.

In the literature there is often a weight loss of 5% or 10% chosen to define cachexia. Therefore we chose 5% and 10% to demonstrate the changes in results, when weight loss is 10% or more. In patients suffering from weight loss exceeding 5% there is already a significant reduced resection rate to detect, but there are not yet differences seen in laboratory parameters and lung function. But when weight loss is 10% or more, there are many more significant changes seen: first of all a significant reduced resection rate is obvious, as there are significantly more often metastases diagnosed; but furthermore there are dramatic changes in laboratory results, amount of fat and muscle tissue and lung function to detect. These findings confirm the fact that a weight loss of 10% has a more significant systemic effect in patients with pancreatic cancer and not 5% but 10% should be chosen as the divison parameter. But nevertheless, the symptom "weight loss" in a patient with pancreatic cancer should make one think of more progressed disease.

The multivariate analysis performed revealed tumour stage and weight loss as significant factors influencing survival in patients with pancreatic cancer.

## Conclusion

In conclusion, we showed that nearly 40% of patients with pancreatic cancer who present for tumour resection suffer from weight loss of 10% or more of their stable pre-illness weight. Due to metastases to the liver and/or peritoneal cavity, the resection rate is significantly reduced in patients with cachexia. A dramatic weight loss in a patient with pancreatic cancer may hint at a more progressed or more aggressive tumour.

A weight loss of 10% had a significant systemic effect, which was not as strong as for a 5% weight loss; the resection rate was also significantly worse when weight loss exceeded 5%, but the nutritional status showed significant differences only when the division parameter was weight loss of 10%.

The most obvious systemic effect of cachexia in pancreatic cancer are the following: 1) a higher rate of more progressed tumour stages in patients with cachexia, 2) a worse nutritional status, indicated by lower protein, albumin, and haemoglobin levels, 3) impaired lung function, and 4) the reduction of fat tissue. Overall, patients with cachexia and pancreatic cancer had significantly reduced survival, and in patients undergoing curative resection even a weight loss of 5% showed significant differences. After detection of metastases, only a preoperative weight loss of 10% worsened survival.

As weight loss of 10% in patients with pancreatic cancer produces more dramatic effects and changes in preoperative laboratory results and the postoperative course, this should be the essential division parameter in pancreatic cancer patients.

## Competing interests

The authors declare that they have no competing interests.

## Authors' contributions

JB, KK, CM and KF collected the patient data and performed the follow-up. CM collected lung function parameters; CM and KF performed the measurements of the CT scans. JB, KK, CM, KF, HKR, and MEM participated in the design of the study. JB, KK and MEM performed the statistical analysis. JB and KK wrote the manuscript. HF, MWB and MEM conceived of the study, participated in its design and coordination, and helped prepare the manuscript. Every author has read and approved the manuscript.

## Pre-publication history

The pre-publication history for this paper can be accessed here:

http://www.biomedcentral.com/1471-2407/9/255/prepub

## References

[B1] Carpelan-Holmstrom M Nordling S Pukkala E Sankila R Luttges J Kloppel G Haglund C Does anyone survive pancreatic ductal adenocarcinoma? A nationwide study re-evaluating the data of the Finnish Cancer Registry Gut 2005 54 385 387 15710987 10.1136/gut.2004.047191 PMC1774412

[B2] Friess H Isenmann R Berberat P Kleeff J Buchler MW [Prognosis in pancreatic carcinoma] Ther Umsch 1996 53 5 401 407 8685859

[B3] Jemal A Murray T Ward E Samuels A Tiwari RC Ghafoor A Feuer EJ Thun MJ Cancer statistics CA Cancer J Clin 2005 55 10 30 10.3322/canjclin.55.1.10 15661684

[B4] Beger HG Rau B Gansauge F Poch B Link KH Treatment of pancreatic cancer: challenge of the facts World J Surg 2003 27 1075 1084 10.1007/s00268-003-7165-7 12925907

[B5] Conlon KC Klimstra DS Brennan MF Long-term survival after curative resection for pancreatic ductal adenocarcinoma. Clinicopathologic analysis of 5-year survivors Ann Surg 1996 223 273 279 8604907 10.1097/00000658-199603000-00007 PMC1235115

[B6] Cooperman AM Pancreatic cancer: the bigger picture Surg Clin North Am 2001 81 557 574 10.1016/S0039-6109(05)70143-2 11459271

[B7] Imamura M Doi R Imaizumi T Funakoshi A Wakasugi H Sunamura M Ogata Y Hishinuma S Asano T Aikou T Hosotani R Maetani S A randomized multicenter trial comparing resection and radiochemotherapy for resectable locally invasive pancreatic cancer Surgery 2004 136 1003 1011 10.1016/j.surg.2004.04.030 15523393

[B8] Kleeff J Michalski C Friess H Buchler MW Pancreatic cancer: from bench to 5-year survival Pancreas 2006 33 111 118 10.1097/01.mpa.0000229010.62538.f2 16868475

[B9] Kleeff J Michalski CW Friess H Buchler MW Surgical treatment of pancreatic cancer: The role of adjuvant and multimodal therapies Eur J Surg Oncol 2007 33 817 823 17331695 10.1016/j.ejso.2007.01.022

[B10] Wagner M Redaelli C Lietz M Seiler CA Friess H Buchler MW Curative resection is the single most important factor determining outcome in patients with pancreatic adenocarcinoma Br J Surg 2004 91 586 594 10.1002/bjs.4484 15122610

[B11] Freelove R Walling AD Pancreatic cancer: diagnosis and management American family physician 2006 73 485 492 16477897

[B12] Dank M [Tumorus anorexia/cachexia syndrome] Magy Onkol 2001 45 431 436 12050692

[B13] DiMagno EP Pancreatic cancer: clinical presentation, pitfalls and early clues Ann Oncol 1999 10 140 142 10.1023/A:1008382029375 10436807

[B14] Ockenga J Pirlich M Gastell S Lochs H Tumour anorexia–tumour cachexia in case of gastrointestinal tumours: standards and visions Zeitschrift fur Gastroenterologie 2002 40 929 936 10.1055/s-2002-35411 12436371

[B15] Deans C Wigmore SJ Systemic inflammation, cachexia and prognosis in patients with cancer Curr Opin Clin Nutr Metab Care 2005 8 265 269 15809528 10.1097/01.mco.0000165004.93707.88

[B16] Davidson W Ash S Capra S Bauer J Cancer Cachexia Study Group Weight stabilisation is associated with improved survival duration and quality of life in unresectable pancreatic cancer Clin Nutr 2004 23 239 247 10.1016/j.clnu.2003.07.001 15030964

[B17] Costelli P Baccino FM Mechanisms of skeletal muscle depletion in wasting syndromes: role of ATP-ubiquitin-dependent proteolysis Curr Opin Clin Nutr Metab Care 2003 6 407 412 10.1097/00075197-200307000-00009 12806214

[B18] Falconer JS Fearon KC Ross JA Elton R Wigmore SJ Garden OJ Carter DC Acute-phase protein response and survival duration of patients with pancreatic cancer Cancer 1995 75 2077 2082 10.1002/1097-0142(19950415)75:8<2077::AID-CNCR2820750808>3.0.CO;2-9 7535184

[B19] Fearon KC Barber MD Falconer JS McMillan DC Ross JA Preston T Pancreatic cancer as a model: inflammatory mediators, acute-phase response, and cancer cachexia World J Surg 1999 23 584 588 10.1007/PL00012351 10227928

[B20] Tisdale MJ Cancer cachexia Langenbecks Arch Surg 2004 389 299 305 10.1007/s00423-004-0486-7 15168125

[B21] Bosaeus I Daneryd P Svanberg E Lundholm K Dietary intake and resting energy expenditure in relation to weight loss in unselected cancer patients Int J Cancer 2001 93 380 383 10.1002/ijc.1332 11433403

[B22] Camps C Iranzo V Bremnes RM Sirera R Anorexia-Cachexia syndrome in cancer: implications of the ubiquitin-proteasome pathway Support Care Cancer 2006 14 1173 1183 10.1007/s00520-006-0097-7 16819628

[B23] Agustsson T Ryden M Hoffstedt J van Harmelen V Dicker A Laurencikiene J Isaksson B Permert J Arner P Mechanism of increased lipolysis in cancer cachexia Cancer Res 2007 67 5531 5537 10.1158/0008-5472.CAN-06-4585 17545636

[B24] Inui A Cancer anorexia-cachexia syndrome: are neuropeptides the key? Cancer Res 1999 59 18 4493 4501 10493494

[B25] Laviano A Meguid MM Inui A Muscaritoli M Rossi-Fanelli F Therapy insight: Cancer anorexia-cachexia syndrome–when all you can eat is yourself Nat Clin Pract Oncol 2005 2 158 165 10.1038/ncponc0112 16264909

[B26] Nelson KA Modern management of the cancer anorexia-cachexia syndrome Curr Oncol Rep 2000 2 362 368 10.1007/s11912-000-0031-y 11122866

[B27] Ripamonti C Management of dyspnea in advanced cancer patients Support Care Cancer 1999 7 4 233 243 10.1007/s005200050255 10423049

[B28] Ripamonti C Fusco F Respiratory problems in advanced cancer. Supportive care in cancer: Official journal of the Multinational Association of Supportive Support Care Cancer 2002 10 3 204 216 10.1007/s005200100296 11904785

[B29] Kispert JF Kazmers A Roitman L Preoperative spirometry predicts perioperative pulmonary complications after major vascular surgery Am Surg 1992 58 491 495 1642387

[B30] Sobin LH Wittekind C Sobin LH, Wittekind C Pancreas UICC/TNM classification of malignant tumors 1997 Heidelberg Springer Medizin 87 90

[B31] Wittekind C Meyer HJ Bootz F Pankreas TNM Klassifikation maligner Tumoren 2006 83 85

[B32] Buchler MW Friess H Wagner M Kulli C Wagener V Z'Graggen K Pancreatic fistula after pancreatic head resection Br J Surg 2000 87 883 889 10.1046/j.1365-2168.2000.01465.x 10931023

[B33] Friess H Beger HG Sulkowski U Becker H Hofbauer B Dennler HJ Buchler MW Randomized controlled multicentre study of the prevention of complications by octreotide in patients undergoing surgery for chronic pancreatitis Br J Surg 1995 82 1270 1273 10.1002/bjs.1800820938 7552016

[B34] Ferrone CR Finkelstein DM Thayer SP Muzikansky A Fernandez-delCastillo C Warshaw AL Perioperative CA19-9 levels can predict stage and survival in patients with resectable pancreatic adenocarcinoma J Clin Oncol 2006 24 2897 2902 10.1200/JCO.2005.05.3934 16782929 PMC3817569

[B35] Ortiz-Gonzalez J Alvarez-Aguila NP Medina-Castro JM Adjusted carbohydrate antigen 19-9. Correlation with histological grade in pancreatic adenocarcinoma Anticancer Res 2005 25 3625 3627 16101191

[B36] Rai A Tewari M Mohapatra SC Shukla HS Correlation of nutritional parameters of gallbladder cancer patients J Surg Oncol 2006 93 705 708 10.1002/jso.20539 16724358

[B37] Buchler MW Wagner M Schmied BM Uhl W Friess H Z'graggen K Changes in morbidity after pancreatic resection: toward the end of completion pancreatectomy Arch Surg 2003 138 1310 1314 10.1001/archsurg.138.12.1310 14662530

